# Thymoquinone Could Increase The Efficacy of Tamoxifen Induced Apoptosis in Human Breast Cancer Cells: An In Vitro Study 

**DOI:** 10.22074/cellj.2016.4320

**Published:** 2016-05-30

**Authors:** Sedigheh Ganji-Harsini, Mozafar Khazaei, Zahra Rashidi, Ali Ghanbari

**Affiliations:** Fertility and Infertility Research Center, Kermanshah University of Medical Sciences, Kermanshah, Iran

**Keywords:** Apoptosis, Breast Cancer, Necrosis, Tamoxifen, Thymoquinone

## Abstract

**Objective:**

Thymoquinone (TQ), as the main component of Nigella Sativa plant, shows anticancer properties. This study was aimed to evaluate the combined effect of TQ and Tamoxifen (TAM) on viability and apoptosis of human breast cancer cell lines.

**Materials and Methods:**

In this experimental study, estrogen positive MCF-7 and estrogen negative MDA-MB-231 human breast cancer cell lines were induced by TAM (2 µM) or different doses of TQ (50, 75, 100, 150 µM), individually or in combination. Cell viability and apoptosis were investigated by MTT assay and TdT-mediated deoxy-uracil nick end labeling (TUNEL) assay; Acridine orange (AO)/Ethidium bromide (EB) staining respectively. Data were analyzed by one way ANOVA and P<0.05 was considered significant.

**Results:**

In 24 hours treatment, TAM and all doses of TQ, solely or in combination, significantly reduced cell viability of both cell lines, except in MCF-7 cells treated with 50 µM TQ, and MDA-MB-231 cells treated with 50 or 75 µM TQ (P<0.01). After 48 hours treatment, cell viability of both cell lines was reduced in all treated groups (P<0.05). Remarkable apoptotic index was observed in combination treatment of MCF-7 or MDA-MB-231 cell lines with TAM and TQ (P<0.001).

**Conclusion:**

The synergistic effect of TQ and TAM on human breast cancer cell lines showed cell viability reduction as well as apoptosis induction, independent to estrogen.

## Introduction

As the first line of endocrine therapy, estrogen receptor positive (ER+) breast cancers were treated with Tamoxifen (TAM) (1,2), although low efficacy of this drug for treatment of ER-negative (ER) is still a crucial challenge (3). In addition, resistance to this therapeutic agent is the other problem reducing the efficiency of TAM therapy. Curiously, evidences showed that TAM acts as a chemical structure -like estrogenin chronic treatments, promoting progression of the tumors (4). 

Another limitation at treatment of breast cancer cells with TAM is that the long-time administration of this agent leads to transforming growth factor-β (TGF-β) over-expression. TGF-β contributes to induction and maintenance of many cellular processes such as proliferation, apoptosis, migration and angiogenesis. It has been shown that high level of TGF-β, at the first stage of breast cancer formation, declined progression of the tumors. However, following on tumor progression, this growth factor could contrarily induce metastasis, leading to distribution of the cancer (5,7). 

Combination therapy application with medicinal plants, which cause little side-effects, is one of the current strategies to improve the efficiency of TAM treatment in breast cancer patients (8). Thymoquinone (TQ) is a principle compound of Nigella Sativa or Black Seed (a medicinal plant), acting as an anti-allergic, anti-oxidant, anti-diabetic and also anti-tumor substance (9). Investigations revealed that this compound mainly increased the rate of malignant cell apoptosis *in vitro*, solely or in combination with anti-cancer agents like Cisplatin, while the side-effects on the normal tissues (e.g. kidney and heart) were reduced *in vivo* (9,11). Interestingly, it was proposed that TQ could potentially be administrated as an anti-cancer drug in future (12). It was later demonstrated that TQ promoted apoptosis in breast cancer cells through X-linked inhibitor of apoptosis protein (XIAP) mediated protein kinase B (PKB), also known as Akt pathway (13). 

With regards to the poor outcome of current chemotherapies on breast cancer treatment, improvement of this approach looks necessary. Although TQ has solely been proposed as an anti-cancer compound, some evidences showed application of this drug in combination with TAM, in the literatures (13). The aim of present study was to evaluate the efficacy of TAM to induce apoptosis and inhibit proliferation in bot ER+ or ERbreast cancer cell lines, in combination with TQ. 

## Materials and Methods

This experimental *in vitro* study was conducted in Fertility and Infertility Research Center at Kermanshah University of Medical Sciences (Kermanshah, Iran). 

### Cell culture and treatment

TAM (Sigma, USA, ALX-550-095-G001) and TQ (Sigma, USA, 274666-1G) agents were individually dissolved in dimethyl sulfoxide (DMSO) at final concentration of 0.1% (v/v). These components were applied individually or in combination for treatment of breast cancer cell lines. A single dose of 2 µM TAM was individually used for treatment. In addition, different doses of 50, 75, 100, 150 µM TQ were utilized individually or in combination with TAM. 

### Cell viability assay to determine optimal dose and time course for Thymoquinone

Estrogen positive (MCF-7) and estrogen negative (MDA-MB-231) human breast cancer cell lines were cultured at 37˚C in a humidified incua bator containing 5% CO_2_ . The culture medium was composed of Roswell Park Memorial Institute 1640 (RPMI1640, Gibco, Australia) with 10% fetal calf serum (Sigma, USA) and penicillin/streptomycin (Sigma, USA) antibiotics. Approximately 104 cells were grown in each well of 96-well culture plates and incubated for 24 hours, followed by different dosages and patterns of drug treatment, three times. 

Briefly, the cells were induced by TAM (2 µM) (14) and different doses of TQ (50, 75, 100, 150 µM) in the 96-well plates, followed by measuring cell viability by MTT assay (Sigma, USA, M2003) after 24 and 48 hours. In this experiment, 20 μl of MTT solution [5 mg/ml in phosphate buffer saline (PBS, Merck, Germany)] was added to each well and incubated for 4 hours at 37˚C. The medium with MTT were then removed, and 100 μl DMSO was added to dissolve Formazan-crystal, followed by incubation at room temperature for 30 minutes. The optical density (OD) of each well was ultimately measured by ELISA plate reader (stat fax100, USA) at 570 nm. 

### Cell morphological analysis

Two MCF-7 and MDA-MB-231 cell lines were treated with individual TAM (2 µM) or TQ (150 µM) agent as well as combination of them either during 24 or 48 hours in 96-well plates in three biological experiments. The wells were subsequently prepared for double fluorescent staining with Acridine orange (AO)–Ethidium bromide (EB). In fact, AO staining agent is observed in the survived and dead cells. By binding with double strand DNA, it induces green fluorescence in living cells, while due to binding with single strand DNA, dominantly observed in dead cells, AO induces red fluorescence. EB was excluded from living cells. Although both late apoptotic and necrotic cells have ruptured membrane, permitting EB to enter into the cells and interact with DNA, the former and later cells show sharp and pale staining, respectively. Thus, live cells will show a normal green nucleus. Early apoptotic cells give bright green nucleus with condensed or fragmented chromatin. Late apoptotic cells display condensed and fragmented orange chromatin and necrotic cells have a structurally normal orange nucleus. 

After incubation, the cells were detached with 0.25% trypsin–EDTA (Sigma, USA) and washed once with PBS. Subsequently, 10 µl of the cells were put on a glass slide and mixed with 10 µl AO (50 mg/ml) and EB (50 mg/ml). The cells were analyzed under a fluorescence microscope (Leica, Germany) with ×200 magnification. Percentage of apoptotic and necrotic cells per total number of the cells were counted under a fluorescence microscope in five random fields, average numbers of which were considered as apoptotic or necrotic index. 

### TdT-mediated deoxy-uracil nick end labeling assay

TUNEL assay was carried out using in situ Cell Death Detection Kit, AP (Roche Diagnostics, Germany) according to the manufacturer’s instructions. Briefly, after 48 hours treatment with TAM (2 µM), TQ (150 µM) or combination of them, the cells were fixed by adding 4% paraformaldehyde (Merck, Germany) and incubating for 30 minutes. The fixed cells were washed in PBS, permeabilized with 0.1% Triton X-100 (Sigma, USA) for 5 minutes on ice, and then incubated with 50 μl of terminal deoxynucleotidyl transferase end-labeling solution for 60 minutes at 37˚C in a humidified chamber in dark. The cells were then counterstained in propidium iodide (PI, Sigma, USA) staining solution for 4 minutes at room temperature in dark. The percentage of positively stained cells per total number of the cells was ultimately counted under a fluorescence microscope in five random fields and the average numbers were considered as apoptotic index. 

### Statistical analysis

Statistical analysis was conducted using oneway analysis of the variance (ANOVA) and Tukey’s test. All statistical analyses were done using SPSS software (version 19.0). In all cases, P<0.05 was considered significant. 

## Results

### Cytotoxic effect of Tamoxifen and/or Thymoquinone on breast cancer cells

**Fig.1:**
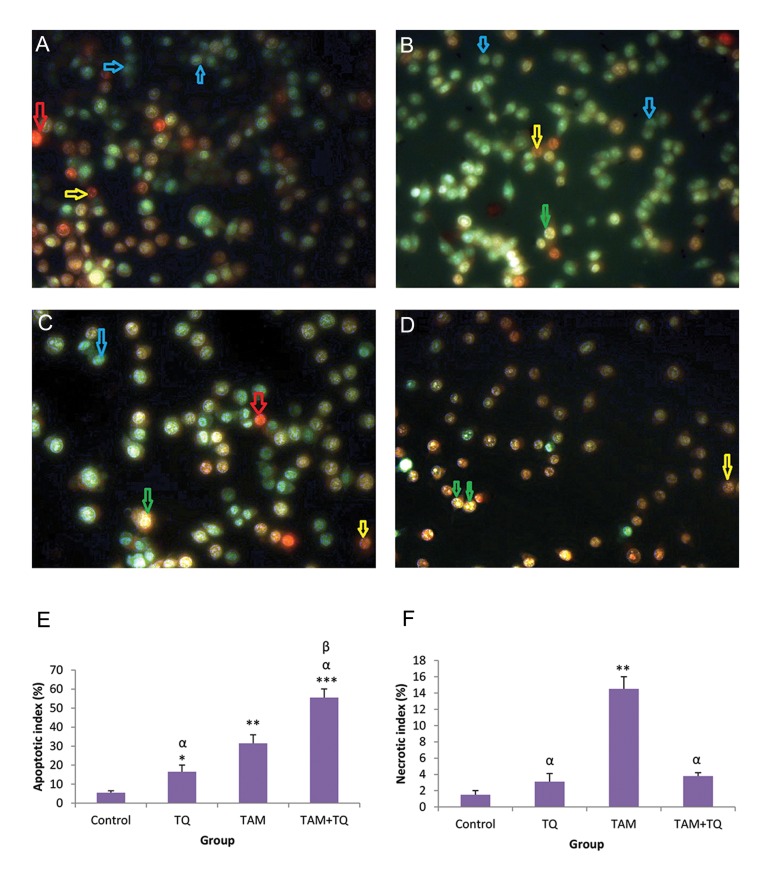
The effects of individual TAM (2 µM) and TQ, or in combination, on viability in MCF-7 and MDA-MB-231 cells. Cells were treated with TAM, TQ and combination of both for A., B. 24 hours, C. and D. 48 hours. Control wells were treated with equivalent amount of agents. Treatment with combined TAM and TQ significantly decreased cell viability compared to individual agents. The results are shown as the mean ± SEM from triplicate experiments. TAM; Tamoxifen, TQ; Thymoquinone, ***; P<0.001, **; P<0.01, *; P<0.05 compared to control, α; P<0.05 compared to TAM and β; P<0.001 in comparison with TAM.

Cytotoxic effect of TAM and TQ, alone or in combination, on cell viability was evaluated by MTT assay in both MCF-7 and MDAMB-231 cell lines (Fig.1). Data obtained from 24 hours treatment of MCF-7 showed significant reduction in cell viability in all experimental groups, except for those treated with 50 µM TQ (Fig.1A). Compared to TAM, cell viability was significantly reduced in combination treatments with TAM+TQ (50 µM), TAM+TQ (75 µM), TAM+TQ (100 µM) and TAM+TQ (150 µM). In addition, MCF-7 cell viability was reduced in all individual as well as synergistic groups compared to treatment with TAM, after 48 hours (Fig.1B). 

Following on 24 hours treatment of MDAMB-231 cells, cell viability was significantly reduced in TAM, TQ (100 µM, 150 µM), and all synergistic groups (Fig.1C). In comparison with TAM, significantly decreased cell viability was observed in TQ (150 µM) and all synergistic treatment groups. After 48 hours treatment, cell survival of MDA-MB-231 was significantly reduced in all experimental groups compared to the control and TAM groups (Fig.1D). With regards to nontoxicity and less cell viability with 48 hours of induction, dosage of 150 µM TQ was selected for further studies. 

### Detection of cell death in MCF-7 and MDAMB-231

**Fig.2:**
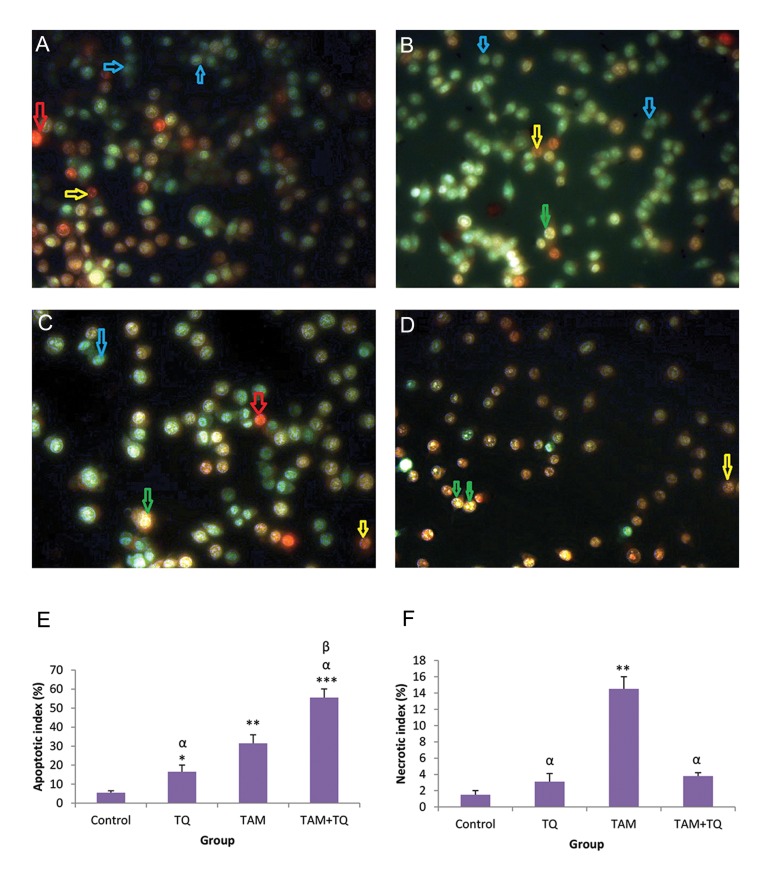
The effects of TAM and TQ alone, or in combination, on morphology of MCF-7. The cells were stained by AO-EB and observed under fluorescence microscope. Images show A. Control group, B. In the presence of 2 µM TAM, C. In the presence of 150 μM TQ, D. In combination of both agents, E. Apoptotic index and F. Necrotic index based on AO-EB stained cells. Arrows show different cell morphologies: Blue; Live, Green; Early apoptotic, Yellow; Late apoptotic and Red; Necrotic cells. Microscopic images were captured with ×200 magnification. TAM; Tamoxifen, TQ; Thymoquinone, AO-EB; Acridine orange–Ethidium bromide, ***; P<0.001, **; P<0.01, *; P<0.05 compared to control, α; P<0.05 compared to TAM, and β; P<0.01 compared to TQ group. The data are presented as mean ± SEM.

**Fig.3:**
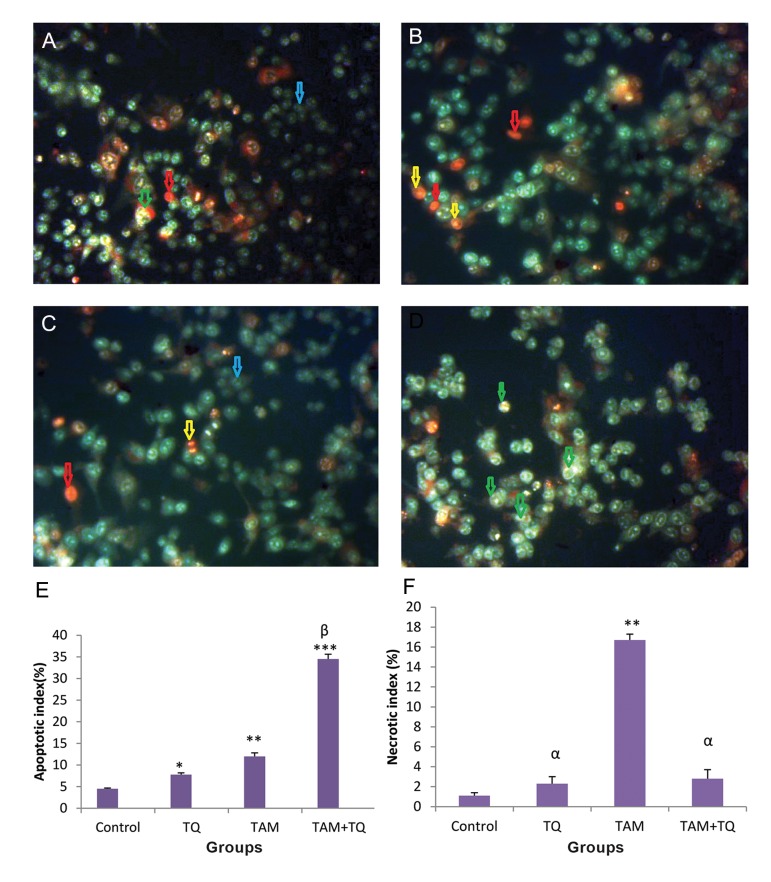
The effects of TAM and TQ alone, or in combination, on morphology of MDA-MB-231. The cells were stained by AO-EB and observed under fluorescence microscope. Images show A. Control group, B. In the presence of 2 µM TAM, C. In the presence of 150 µM TQ, D. In combination of both agents, E. Apoptotic index and F. Necrotic index based on AO-EB stained cells. Arrows show different cell morphologies; Blue; Live, Green; Early apoptotic, Yellow; Late apoptotic and Red; Necrotic cells. Microscopic images were captured with ×200 magnification. TAM; Tamoxifen, TQ; Thymoquinone, AO-EB; Acridine orange–Ethidium bromide, ***; P<0.001, **; P<0.01, *; P<0.05 compared to control, α; P<0.05 compared to TAM group and β; P<0.001 compared to TAM and TQ groups. The data are presented as mean ± SEM.

In this experiment, apoptosis stages were detected in both MCF-7 and MDA-MB-231 cells treated with individual TAM or TQ agent as well as in combination, by AO–EB staining (Fig.2,3). Morphological characteristics of dead cells in MCF-7 showed both early and late apoptosis in TAM and 150 μM TQ treated groups, while only early apoptosis was determined in almost all synergistic groups (Fig.2A-D). 

In MDA-MB-231, both early and late apoptotic cell morphologies were detected in TAM and 150 μM TQ treated groups (Fig.3B,C). In combination of TAM and TQ 150 μM, some MDA-MB-231 cells were detected in early and some others in late stages of apoptosis; although the outbreak of the former stage was lower (Fig.3D). 

According to the data obtained from fluorescent staining of MCF-7 cells with AO-EB, apoptotic index was significantly increased in TQ 150 µM (P<0.05), TAM (P<0.01), TQ 150 µM+TAM groups (P<0.001) in comparison with control group (Fig.2E). Moreover, apoptotic index in TQ150 µM+TAM was significantly increased, in comparison with TAM (P<0.05) and TQ (P<0.01), indicating synergistic effect of TQ on apoptotic induction of TAM in the MCF-7 cells. This index was significantly increased in MDA-MB-231 cells treated with TQ 150 µM (P<0.05), TAM (P<0.01) or TQ 150 µM+TAM groups (P<0.001) in comparison with control (Fig.3E). In addition, apoptotic index was significantly increased in TQ150 µM+TAM compared to TAM and TQ (P<0.001), suggesting synergistic effect of TQ on apoptotic induction of TAM in the MDA-MB-231 cells. 

The necrotic index was also evaluated in MCF-7 (Fig.2F) and MDA-MB-231 cells (Fig.3F). In comparison with control group, the necrotic index was significantly increased in TAM group (P<0.01) while no significant change was observed by treatment of both cell lines with TQ and TAM+TQ groups. Moreover, the necrotic index was significantly reduced in TQ and TQ+TAM groups compared to TAM (P<0.05) in both treated cell lines. 

### TdT-mediated deoxy-uracil nick end labeling assay 

**Fig.4:**
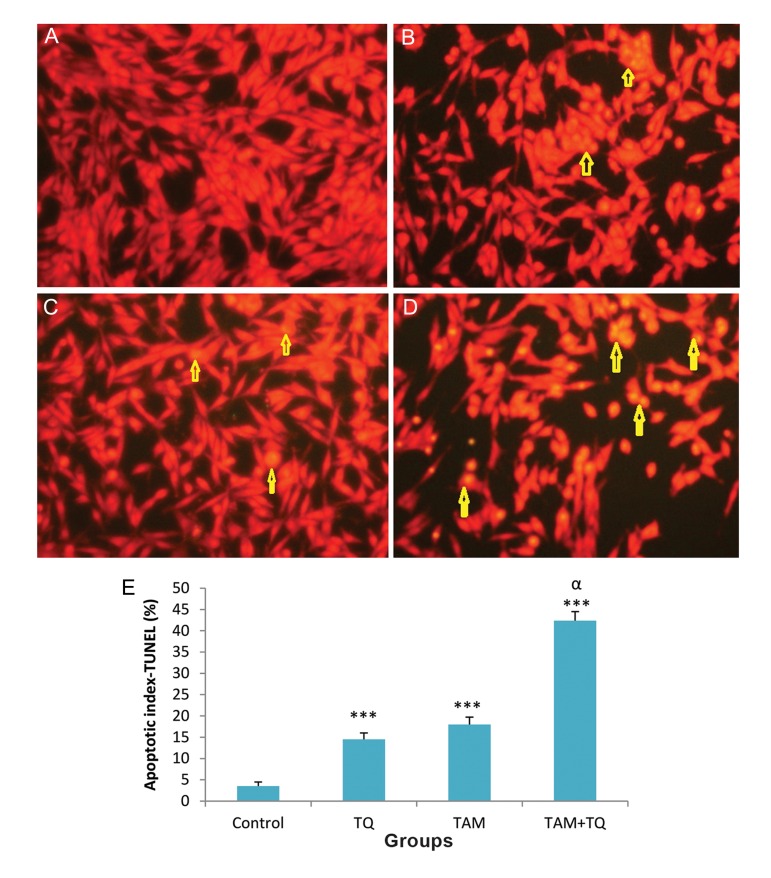
Terminal deoxynucleotidyltransferase-mediated dUTP nick end labeling (TUNEL) staining assay for apoptosis in MCF-7 cells following treatment with TAM and TQ alone, or in combination. A. Control group, B. In the presence of 2 µM TAM, C. In the presence of 150 µM TQ, D. In combination of both agents and E. Apoptotic index, columns show mean percentage of apoptotic cells from three independent experiments performed in triplicate at images. Microscopic images were captured with ×200 magnitudes. TAM; Tamoxifen, TQ; Thymoquinone, ***; P<0.001 compared to control group, α; P<0.01 compared to TAM and TQ groups. The data are presented as mean ± SEM.

TUNEL assay (15) was performed to establish the data of AO-EB staining. This method is simple and sensitive for the detection of apoptotic cells. TUNEL staining clearly displayed apoptotic cells in MCF-7 and MDA-MB-231 cells treated with individual TAM or TQ and combination of them compared to untreated control cells (Fig.4A,B,5A,B). The numbers of apoptotic cells were quantified and presented as percentages (Fig.4C,D,5C,D). Following on combined treatment of TAM and TQ for 48 hours, apoptotic index was significantly increased in both MCF-7 and MDA-MB 231 cell lines (P<0.001, Figes.4E, 5E). 

**Fig.5:**
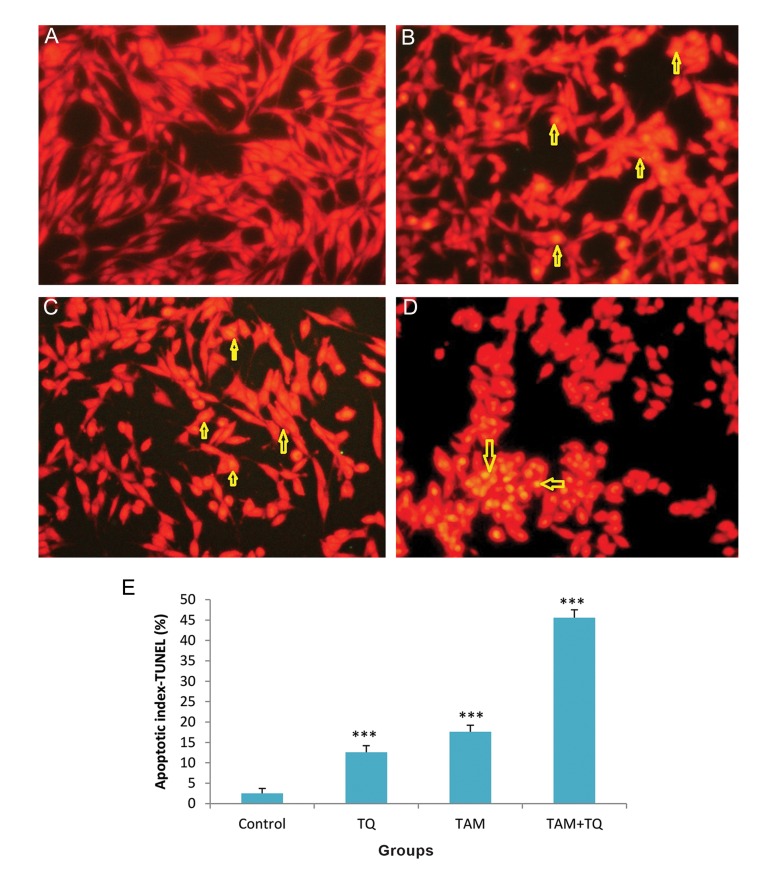
Terminal deoxynucleotidyltransferase-mediated dUTP nick end labeling (TUNEL) staining assay for apoptosis in MDA-MB-231 cells following treatment with TAM and TQ alone, or in combination. A. Control group, B. In the presence of 2 µM TAM, C. In the presence of 150 µM TQ, D. In combination of both agents and E. Apoptotic index, columns show mean percentage of apoptotic cells from three independent experiments performed in triplicate at images. Microscopic images were captured with ×200 magnitudes. TAM; Tamoxifen, TQ; Thymoquinone, ***; P<0.001 compared to control group, α; P<0.01 compared to TAM and TQ groups. The data are presented as mean ± SEM.

## Discussion

The present study indicated that TAM effect was enhanced in combination with TQ, suggesting combination of these two agents produce a significant additive cytotoxic effect in both cell lines. These findings also demonstrated that TAM and TQ inhibited MCF-7 and MDA-MB-231 cells proliferation by inducing apoptosis due to the synergistic effect of the combination treatment. 

Resistance to TAM treatment for both ER+ and ERmetastatic breast cancer tumors is a limitation of this anti-cancer drug. Thus, 50% of the patients do not respond to the drug in long-term treatment. Hence, it is essential to achieve the best dose and period of TAM treatment to reduce the size of the tumor (4). Thus far, the molecular mechanism(s) involved in TAM treatment is not completely understood. Several metabolic and pharmacokinetic mechanisms are regulated in TAM treated cancer cells, leading to accumulation of the drug in the cell micro-organelles as well as changes in several gene expressions and protein syntheses, including β-tubulins, topoisomerase II, DNA repairs, and changes in apoptotic pathways (16,17). 

Disabling apoptosis is a central event in tumorigenesis. In contrast, most chemotherapeutic drugs require apoptotic pathway functions to induce cell death (18). Through both classical and non-classical mechanisms, estrogen results in a general upregulation of genes involved in cell proliferation and survival, as well as down-regulation of genes contributing to anti-proliferative or pro-apoptotic activity, leading ultimately to growth stimulation and apoptosis suppression (19). Therefore, through direct interaction with the ER, anti-estrogens are able to decrease cancer cell proliferation by indirectly regulating the cell cycle and cell death signaling pathways (20). It has been demonstrated that after long-term treatment of breast cancer cells with anti-estrogens, mitochondria-mediated apoptosis could be increased (21). Consequently, it was reported that low concentration TAM treatment induced cell-cycle arrest (22) leading to blocking cancer cells in G0/G1 phase (21). Investigations also indicated that administrating pharmacological concentrations of this drug induced apoptosis in breast cancer cells (23). 

In present study, synergistic effect of TQ and TAM agents on both ER+ and ERcells showed that TQ function trough non-estrogenic dependent pathway. Investigations revealed that TAM induced apoptosis in ER+ cells via nitric oxide (NO)–dependent pathways (24). TAM blocked telomerase activity could also result in apoptosis of tumor cells (25). 

Recently, it is been shown that TQ induces synergistic effect on TAM via XIAP mediated Akt regulation in both ER+ and ERbreast cancer cells (13). Herein, we demonstrated that higher doses of TQ, in combination with low dose of TAM (2 µM), could induce similar results to the function of 5 µM TAM induction (13). The present study, for the first time, indicated that treatment of breast cancer cells with high doses of TQ (alone or in combination with TAM) increased apoptosis and necrosis. We also reported that combination therapy could lead to early apoptosis. This data indicate that high doses of TQ never trigger necrosis process in the breast cancer cells. In this context, Shoieb et al. (26) and Das et al. (27) studies previously showed that administration of this natural component had no side-effects. However, further investigations are required to validate this effect on mammary normal cells. Moreover, we demonstrated that TQ anti-tumor action is apart from estrogen pathway, confirming previous Farah and Begum (28), Effenberger et al. (8) and El-Aziz et al. (29) reports. 

## Conclusion

This study indicates that using high doses of TQ could reduce treatment of both ER+ and ERbreast cancer cells with high TAM concentration and administration time-course. Since the number of necrotic cells is not increased in TQ treated groups, this study may present a safe and non-hazardous approach for the treatment of resistant metastatic breast cancer patients. 
